# Gene expression in local stroma reflects breast tumor states and predicts patient outcome

**DOI:** 10.1038/srep39240

**Published:** 2016-12-16

**Authors:** Russell Bainer, Casey Frankenberger, Daniel Rabe, Gary An, Yoav Gilad, Marsha Rich Rosner

**Affiliations:** 1 Department of Human Genetics, University of Chicago, Chicago, IL 60637, USA; 2 Ben May Department for Cancer Research, University of Chicago, Chicago, IL 60637, USA; 3 Department of Surgery, University of Chicago, Chicago, IL 60637, USA

## Abstract

The surrounding microenvironment has been implicated in the progression of breast tumors to metastasis. However, the degree to which metastatic breast tumors locally reprogram stromal cells as they disrupt tissue boundaries is not well understood. We used species-specific RNA sequencing in a mouse xenograft model to determine how the metastasis suppressor RKIP influences transcription in a panel of paired tumor and stroma tissues. We find that gene expression in metastatic breast tumors is pervasively correlated with gene expression in local stroma of both mouse xenografts and human patients. Changes in stromal gene expression elicited by tumors better predicts subtype and patient survival than tumor gene expression, and genes with coordinated expression in both tissues predict metastasis-free survival. These observations support the use of stroma-based strategies for the diagnosis and prognosis of breast cancer.

Specific molecular interactions between cancer and the various stromal cells within the tumor microenvironment enable tumor invasion, intravasation, and metastasis at distant sites^1^. Metastatic tumors disrupt the tissue homeostasis that maintains the integrity of borders between distinct cell populations in adult tissues as a component of malignant progression^2^. A key part of this process is the dynamic remodeling of the microenvironment as tumors selectively recruit and reprogram stromal cells to facilitate invasion^3^. Consequently, gene expression profiles in tumor-adjacent stromal tissue are influenced by a combination of cell autonomous effects and structural changes that reflect tumor aggressiveness^3^,^4^.

The clear clinical relevance of transcriptional states within the tumor microenvironment has prompted extensive study of stroma composition and transcriptional properties in an effort to understand their relationship to tumor biology. Often, these studies have focused on physically or computationally deconvolving stroma gene expression profiles to identify cell types that play important roles in particular tumor contexts^5^,^6^,^7^. Alternatively, in cases where causal relationships are already known, *in vitro* co-culture studies have been used to identify transcriptional changes induced by the presence of specific cell types^8^,^9^,^10^. While useful for clarifying individual mechanisms within a particular microenviroment, these approaches cannot be practically extended to understand the variability in tumor-stroma relationships across lesions *in vivo*. They also focus on understanding properties of individual stromal components instead of the more complex tissue-level states that influence tumor phenotypes at a larger scale^11^,^12^,^13^.

Here, we use a set of isogenic breast cancer models with different metastatic phenotypes to characterize the extent to which tumor and bulk stroma gene expression levels are coordinated across tumor–stroma boundaries. We show that tumor and stroma gene expression levels are largely positively correlated. In addition, we find that a smaller fraction of genes whose expression is negatively correlated between tumor and host tissues has clinical utility for the diagnosis and prognosis of malignant breast tumors.

## Methods

### Study Design and Xenograft Experiments

All animal work was done in accordance with a protocol approved by the Institutional Animal Care and Use Committee. We injected one rear mammary fat pad from each of five athymic nude mice with MDA-MB-231-derived BM1 breast carcinoma cells suspended in PBS and allowed tumors to develop for four weeks. We then sacrificed the mice and extracted both the tumor-bearing and uninjected fat pads, bisected each fat pad, and separately isolated RNA from each tissue section. We then sequenced these RNA samples using the Illumina HISEQ2000 platform and separated the mouse and human reads to generate tumor-specific and stroma-specific gene expression estimates. We compared these estimates with similarly-derived expression estimates from fat pads taken from mice injected with BM1 cells stably expressing the RKIP metastasis suppressor at physiological levels, and from mice that were sham injected and do not contain developing tumors. This approach allows us to identify both local and systemic changes in stromal mRNA expression related to the presence of developing invasive and noninvasive tumors, and to identify changes in tumor expression *in vivo* without the confounding effect of transcription from infiltrating host cells. The study design is summarized in Supplementary Fig. 1. Further details about species-specific alignment, quality control, expression estimation, and computational modeling are provided in the supplementary material.

All confirmation experiments were performed in separate panels of xenograft mice that were generated identically to those in the RNAseq experiment. Where appropriate, expression levels in combined human and mouse tissue samples were validated using species-specific RT-PCR primers; a list of these primer sequences is included in the supplementary materials.

### Coexpression Network Analysis

Individual gene ortholog pairs in human and mouse were identified using the Homologene database release 65 by using the Homologene Matcher tool implemented on the RefDIC database^14^. We performed whole genome coexpression network analysis using the expression estimates of 11,181 genes for which the human gene was unambiguously associated with a single mouse ortholog, and for which the mouse ortholog was unambiguously assigned to the corresponding human gene. We generated two normalized matrices of log-transformed FPKM gene expression estimates from the set of samples for which both tumor and stroma expression estimates were available, maintaining the sample order between mouse-specific and human specific matrices. We then independently generated tumor and stroma coexpression networks from the gene data, and extracted the eigengenes from each of the resulting modules. We subsequently generated a correlation matrix using these eigengenes, and identified pairs of coexpressed modules based on the pairwise Pearson’s correlation coefficients generated by comparing the eigengenes of the mouse and human expression modules. All reported module pairs contain disproportionately high numbers of orthologous genes (P = 9.0 × 10^−8^, P = 4.0 × 10^−5^, P = 0.02, Fisher’s exact test), and were not apparently affected by misalignment (correlation coefficients are not well correlated with misalignment rates). We estimated ontological enrichment within the modules using goseq as described, using the set of orthologs present in both modules as the background set.

### Patient Classification by Tumor and Stroma Gene Expression

To determine whether the genes identified in our analysis could be used to improve classification of breast cancer patient samples, we employed a nearest-neighbor clustering approach. First, we hierarchically clustered the patient samples on the basis of their euclidean distance and constructed a dendrogram in which each patient sample is assigned to a leaf. We defined the extent to which samples of a given Pam50 subtype were correctly clustered as the proportion of all nearest-neighbor leaves of all samples in the group that also corresponded to samples of the same Pam50 subtype. Thus, within each dataset each Pam50 subtype was assigned a nearest-neighbor clustering score between zero and one, such that subtypes with a score of one are clustered into a single uninterrupted bloc, and a subtype with a score of zero contains samples that never cluster next to each other. To test the ability of each gene set to improve clustering of a given subtype, we generated empirical *P*-values by comparing the clustering score assigned to that subtype to the null distribution of clustering scores similarly generated by permutation. Further details of this analysis, including a discussion of multiple test correction, are included in the supplement.

### Validation of Coordinated Gene Expression in Human Patients and Evaluation of Clinical Signficance

To test whether gene expression levels are correlated in tumor and stroma tissues derived from human patients we used an expression microarray dataset deposited in the Gene Expression Omnibus by Boersma *et al*.^15^ (GSE5847). The data set consists of 90 microarrays hybridized with RNA from paired tumor and stroma samples isolated via laser capture microdissection from inflammatory and non-inflammatory breast cancer samples. We classified the tumor tissues into basal, HER2 positive, normal, and luminal (combining luminal A and luminal B) subtypes using the PAM50 classifier. We then calculated the Spearman correlation coefficient relating the expression estimate of each probe set in the tumor and stroma tissues, and plotted the distribution of these values for all samples or separately within only the basal and luminal patient subsets.

To determine if the expression levels of genes in a set were prognostic for metastasis-free survival in human patient data, we used the rsf function implemented in the randomSurvivalForest R package to assign variable importance scores to each gene in that set on the basis of their expression within the human breast cancer data set. Then, we combined all genes with positive variable importance scores into a classifier which we used to subdivide the patient samples into two groups via k-means clustering. Finally, we determined whether there was a relationship between group assignment and metastasis-free survival by fitting a cox proportional hazards model to the patient data and including histological subtype and patient age as covariates. We tested the significance of the association in the framework of the model using a Wald test^16^.

## Results

**Figure 1:**
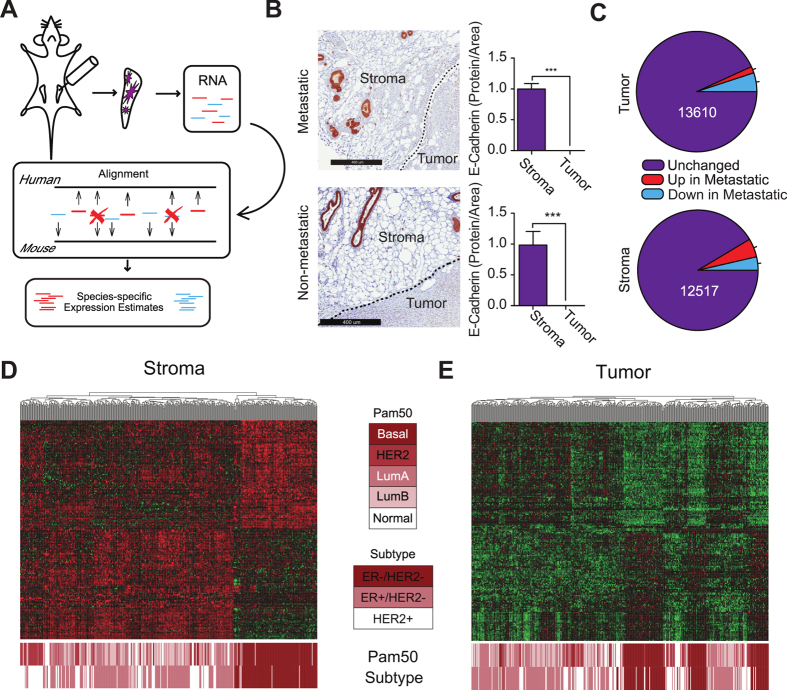
Stromal genes classify better than tumor. (**A**) Scheme illustrating separation of sequence reads derived from whole mammary fat pads containing developing tumors on the basis of species origin. Human- and mouse-derived reads are shown in red and blue, respectively. (**B**) E-cadherin is exclusively expressed in the tumor stroma of both metastatic and non-metastatic tumors, as indicated by representative immunohistochemical staining (top and bottom left panels). Protein expression of E-cadherin was quantified as the proportion of tumor or tumor stroma area (top and bottom right panels; n = 4 mice per group). (**C**) Differentially expressed genes (FDR = 0.01) between metastatic (RKIP-) and nonmetastatic (RKIP+) tumors (top) and surrounding stroma (bottom). Human breast tumor samples hierarchically clustered by euclidean distance using all genes significantly differentially expressed between metastatic and nonmetastatic mouse stroma (**D**) and human tumors (**E**) (FDR = 0.01). Heatmaps depict the expression levels of all genes within this set that are nominally differentially expressed between basal and other human tumor samples (P <= 0.05, 2-tailed t-test), with Pam50 and ER/Her2 subtype classifications listed below.

We sequenced RNA derived from a breast xenograft tumor model and used a multispecies alignment approach to resolve human tumor and mouse stromal transcripts^17^,^18^,^19^,^20^,^21^ (Fig. 1A and Supplementary Methods). The metastatic BM1 human breast tumor cells used were derived from basal/triple-negative breast cancer (TNBC) that is generally associated with poorer patient prognosis, in contrast to the luminal subtype^22^. We focused on gene expression changes specific to metastasis by comparing isogenic metastatic tumors to nonmetastatic tumors that stably express the metastasis suppressor Raf Kinase Inhibitory Protein (RKIP) (Fig. S1A). This approach enabled us to directly investigate the global effect of a metastatic phenotype on tumor and stroma transcription within the microenvironment, and to determine whether local stromal gene expression provides specific information that may be used to predict patient outcomes.

Species-specific RNAseq has been described previously^21^,^23^, but has primarily been used to eliminate the influence of stromal tissues from xenograft tumor sample expression estimates rather than for joint estimation of tumor and stroma gene expression levels. To validate the method, we demonstrated that single unambiguous species-specific reads could be accurately assigned (Fig. S3). We detected mRNA expressed from 13,337 genes of which 11,662 were expressed in both tumor and stroma, 1391 were expressed exclusively in stroma and 284 were exclusive to the tumor (Fig. S3C). We mapped 84% of the reads to either mouse or human transcriptomes and confirmed by immunohistochemistry that expression of E-cadherin was limited to stroma in both metastatic and nonmetastatic tumors as predicted by RNAseq (Figs 1B and S3D). To corroborate the correspondence between mRNA abundance and the expression of encoded proteins, we compared our estimates of the abundance of extracellular matrix (ECM) proteins to those obtained in a study by Hynes and coworkers^20^, in which ECM components within the tumor microenvironment were assigned using a species-specific proteomics-based approach. We found strong agreement between the expression levels of mRNAs encoding these proteins and their predicted source tissues (Fig. S3E).

Since patient tumor samples typically contain infiltrating stromal cells, gene expression level estimates used to classify clinical samples are derived from a mixture of both tissues. To resolve their relative contribution to patient classification, we compared the accuracy of classifiers derived from tumor and stroma expression estimates. We compared metastatic and RKIP-expressing nonmetastatic tumors to identify genes that stratify tumor tissues (N = 995) and stroma tissues (N = 1176) by their invasive phenotype (Fig. 1C). These two gene sets were independently used to hierarchically cluster microarray data from human breast tumor samples. Genes differentially expressed between metastatic and nonmetastatic stroma tissue effectively classified patients into the basal and TNBC subtypes defined by the Pam50 algorithm^22^ (*P* = 0.003, see Methods). This overall trend was confirmed using two separate human breast cancer gene expression datasets (Figs 1D and S5, S6). Notably, the stroma-derived gene set was uniformly more effective at classifying human tumor samples by subtype than either the tumor-derived gene set or the combined tumor and stroma-derived gene set (Figs 1E and S5, S6).

**Figure 2:**
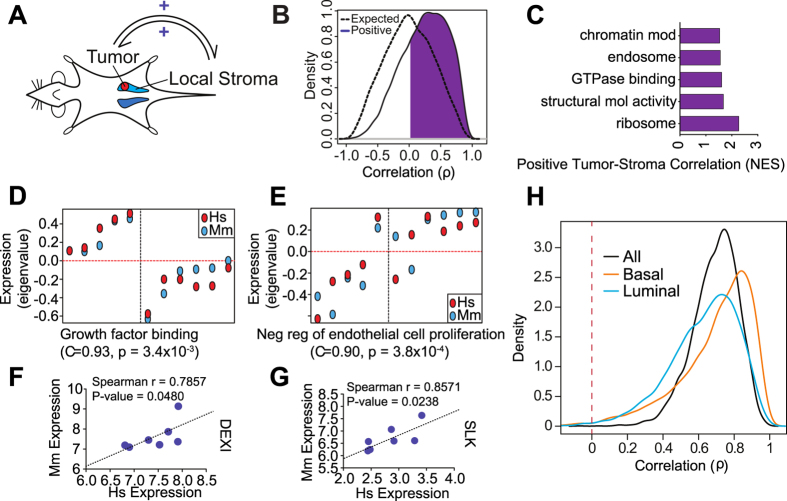
Positive correlation between tumor and local stromal genes observed mouse model and human patients. (**A**) Scheme illustrating the positive feedback between tumor and stroma. (**B**) Density of pairwise Spearman coefficient estimates summarizing the correlation between tumor and stroma mRNA expression levels (purple) for all positively correlated unambiguous gene ortholog pairs. A permutation-derived null distribution is indicated in black. (**C**) Ontological categories most enriched among genes whose ortholog transcription in tumor and stroma is positively correlated (purple) as determined via GSEA, using ortholog expression correlation coefficients as a ranked list, where the enrichment score is indicated along the x-axis. (**D**,**E**) Positively correlated gene expression modules in tumors and adjacent stroma. Weighted correlation network analysis (WGCNA) was independently performed using tumor and stroma expression data and modules with correlated expression patterns in each species identified by inspection. Eigengene expression values (eigenvalues, y-axis) derived from tumor (red) and stroma (blue) modules are indicated for all tumor samples analyzed (x-axis; individual tumors are distributed along the x-axis and stratified by metastatic (RKIP-) and nonmetastatic (RKIP+) tumor type). Module eigengene spearman correlation coefficients and the most enriched ontological category among the genes whose orthologs are present in both modules are indicated below each plot; *P*-values indicate the nominal significance of the ontological enrichment. (**F**,**G**) Validation of correlated gene expression in an independent group of BM1 xenograft tumors via qRT-PCR. Representative genes (DEXI and SLK) from the modules above were assayed with species-specific PCR primer sets, and expression estimates normalized to human GAPDH or mouse Rpl4 are indicated on the x and y axes (ΔΔCt method). Spearman correlation coefficients and corresponding p-values (via permutation) are indicated. (**H**) Validation of high correlation between mRNA expression levels in paired tumor and stroma of samples laser capture microdissected from all (n = 45) (black), luminal (blue) and basal (red) breast cancer tumors.

We reasoned that a microenvironment shared by invasive tumor cells and surrounding stroma could drive common patterns of gene expression in both tissues. To address this possibility, we compared genome-scale estimates of paired tumor and stroma gene expression levels within individual xenograft tumor samples (Fig. 2A). We analyzed 11,181 genes for which single unambiguous species-specific ortholog could be assigned (see Methods). Expression levels of most orthologs (75.6%) were positively correlated between tumor and local stroma across individual tumor samples (Figs 2B and S7A). As expected, gene set enrichment analysis revealed that genes whose expression levels were positively correlated with their stromal orthologs were disproportionately involved in various aspects of cell signaling, protein translation, and chromatin remodeling (Fig. 2C). To further explore the nature of this coordinated expression, genes with similar expression profiles in the tumor and stroma tissue were separated into modules via whole genome co-expression network analysis (WGCNA, see Methods). At least two modules of co-expressed genes were correlated with differences in tumor metastatic state. The first module contained genes largely upregulated in the metastatic microenvironment that were significantly enriched in growth factor binding (*P* = 3.4 × 10^−3^; Fig. 2D). The second module contained genes up-regulated in the nonmetastatic microenvironment and included genes involved in suppressing proliferation of endothelial cells required for angiogenesis and tumor cell dissemination (*P* = 3.8 × 10^−4^; Fig. 2E). We confirmed these observations by species-specific qRT-PCR using representative genes from each of the positively correlated modules (*DEXI/Dexi* and *SLK/Slk,* Fig. 2F,G, *resp.*). The high proportion of genes with positively correlated expression levels in tumor and stroma suggests that a shared microenvironment drives coordinated transcriptional regulation in both tissues.

Notably, we found that gene expression levels in human breast tumors are similarly positively coordinated. We compared expression levels estimated from microarrays hybridized with RNA isolated from paired microdissected human breast tumor and tumor-associated stroma^15^. This correlation is likely to be influenced by genetic differences between patients that jointly influence gene expression levels in both tissues. However, the presence of similar patterns in isogenic xenograft tumor models suggests that a component of the observed correlation is derived from microenvironmental crosstalk. We also found that transcript levels in basal-like tumor samples are better correlated overall with surrounding stroma than transcription in luminal tumors where the tumor and stroma compartments are more distinct^24^. This transcriptional coordination is slightly elevated in inflammatory breast tumors relative to noninflammatory breast tumors, consistent with increased microenvironmental signaling (Fig. 2H and S9). These results clinically validate the predominant positive correlation between tumor and stromal gene expression observed in the xenograft mouse model.

**Figure 3:**
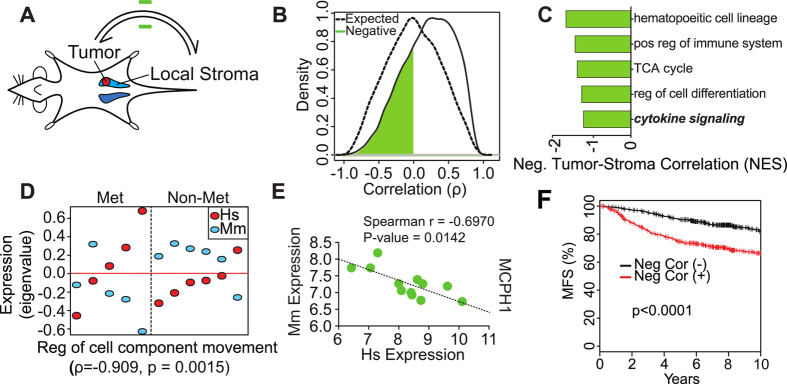
Negative correlation between tumor and local stroma gene expression enables prediction of metastasis-free survival in human patients. (**A**) Scheme illustrating the negative feedback between tumor and stroma. (**B**) Density of pairwise Spearman coefficient estimates summarizing the correlation between tumor and stroma mRNA expression levels (purple) for all negatively correlated unambiguous gene ortholog pairs. A permutation-derived null distribution is indicated in black. (**C**) Ontological categories most enriched among genes whose ortholog transcription in tumor and stroma is negatively correlated (green) as determined via GSEA, using ortholog expression correlation coefficients as a ranked list, where the enrichment score is indicated along the x-axis. (**D**) Negatively correlated gene expression module pair in tumors and adjacent stroma. Weighted correlation network analysis (WGCNA) was independently performed using tumor and stroma expression data and modules with correlated expression patterns in each species identified by inspection. Eigengene expression values (eigenvalues, y-axis) derived from tumor (red) and stroma (blue) modules are indicated for all tumor samples analyzed (x-axis; individual tumors are distributed along the x-axis and stratified by metastatic (RKIP−) and nonmetastatic (RKIP+) tumor type). Module eigengene spearman correlation coefficient and the most enriched ontological category among the genes whose orthologs are present in both modules are indicated below each plot; *p*-values indicate the nominal significance of the ontological enrichment. (**E**) Validation of negatively correlated gene expression in an independent group of BM1 xenograft tumors via qRT-PCR. Representative gene (MCPH1) from the module above was assayed with species-specific PCR primer sets, and expression estimates normalized to human GAPDH or mouse Rpl4 are indicated on the x and y axes (ΔΔCt method). Spearman correlation coefficients and corresponding p-values (via permutation) are indicated. (**F**) Kaplan-Meier plot demonstrating improved prediction of metastasis-free survival (MFS, y-axis) in a cohort of 871 human breast cancer patients by a classifier derived from genes whose expression levels are anticorrelated in tumor and stroma tissue (red line). After stratifying patients on the basis of the classifier, we estimated the significance of the association with MFS in the framework of a Cox multivariate regression model using a Wald test to generate P-values.

We also identified a set of genes whose expression was negatively correlated between tumor and stroma tissues. This set was enriched for genes involved in categories such as cytokine and immune regulation that have been linked to metastatic remodeling of the stroma^3^ (Fig. 3A,B). Among these, WGCNA defined a module pair (eigengene ρ_Pearson_ = −0.96; P < 0.0001) containing genes whose function is related to tissue homeostasis and DNA damage response such as microencephalon (*MCPH1*). These results were independently confirmed in a separate panel of xenografted animals by species-specific qRT-PCR using a representative gene from the negatively correlated module set (*MCPH1/Mcph1*; Fig. 3E).

*MCPH1* functions as a tumor suppressor that regulates mitotic checkpoint activity and *BRCA1*, a DNA repair protein whose loss is associated with the highly metastatic TNBC phenotype^25^,^26^,^27^. We reasoned that negatively correlated genes might disproportionately reflect the stromal response to developing tumors and may therefore provide specific prognostic information in human patients. A gene signature derived from the negatively correlated genes was prognostic for metastasis-free survival (Table S2). This signature successfully stratified patients in multiple human breast cancer gene expression datasets (Figs 3F and S7B). By contrast, a gene signature based on the positively correlated genes did not stratify patients for metastasis-free survival within the same data sets. These results provide clinical validation that genes whose expression is inversely correlated between tumor and local stroma are associated with breast cancer metastasis in humans.

## Discussion

The work presented here demonstrates that metastatic tumors cause coordinated transcriptional changes that prime normal stromal mammary tissue proximal to the site of the primary lesion. This property is a key component of tumor presence and can differentiate metastatic and non-metastatic tumors. We show that gene expression in local tumor and stroma are largely positively correlated. Our results suggest that metastatic tumors effectively co-opt local host physiology, triggering broad transcriptional changes in tissues near the lesion. The transitive propagation of tumor-stromal coordinated gene expression enables genes whose expression is associated with a metastatic phenotype to be diagnostic and prognostic for metastasis-free survival of human breast cancer patients. This phenomenon likely contributes to the metastatic tumor’s ability to disrupt stromal tissue boundaries, enabling extensive remodeling of the stromal compartment and creation of a shared tumor-stromal signaling environment.

Significant previous work has focused on identifying mechanisms by which tumors remodel the local stroma, which sometimes manifest as coordinated gene expression. In a recent paper^28^, we showed that the metastasis suppressor RKIP, through inhibition of the architectural remodeling factor HMGA2, suppresses secretion of CCL5, which in turn recruits a distinct subset of macrophages to TNBC tumors. These macrophages secrete pro-metastatic factors that are also secreted by breast tumor cells and promote tumor invasion. These results illustrate one mechanism by which tumor and local stroma gene expression are positively correlated. Similar positive feedback loops have been described for cancer-associated fibroblasts as well^29^.

Despite clear clinical relevance, the manner in which these various forms of crosstalk combine to form tissue-level states in the surrounding stroma is not well understood. Previous studies comparing paired bulk tumor and stroma samples or isolated tissues have suggested that transcription within the microenvironment may be coordinated by tumor cells, and that elevated expression levels of some genes in the tumor may be reflected in the stroma^4^,^30^,^31^. However, none of these studies could definitively reject technical explanations for this observation. By contrast, the present work supplements our current understanding of microenvironmental interactions by providing direct and complementary evidence that links altered gene expression in tumor-associated stromal tissue states to the transcriptional activity of the corresponding tumor.

By employing a species-specific approach, we are able to rule out trivial explanations for these phenomena to clearly demonstrate that tumor cells influence transcription in endogenous stromal tissues. Our alignment pipeline ensures that we can rule out stromal contamination by disseminated human tumor cells or micrometastases. Furthermore, because we are comparing gene expression across species and tumors in near-isogenic conditions, we can also discount the possibility that genetic differences between individuals account for the commonality in gene expression between tumor and stroma. Finally, while we note that xenograft models imperfectly recapitulate the tumor microenvironment that is present in immune competent animals, our approach has allowed us to definitively observe transcriptional coordination between tumor and stroma tissues, and to make observations that are supported by similar trends in diverse human breast cancer datasets.

The relationship between gene regulatory patterns in tumor cells and local stroma reflects tumor states and therefore has clinical application. The potential predictive nature of stromal transcription within the local tumor microenvironment has been noted previously^4^. However, the direct observation of coordinated gene expression between tumor and stroma cells, both positive and negative, is a novel finding. Importantly, we demonstrate that a subset of these relationships can be used to provide useful diagnostic and prognostic information for human malignancies.

## Additional Information

**How to cite this article**: Bainer, R. *et al*. Gene expression in local stroma reflects breast tumor states and predicts patient outcome. *Sci. Rep.* **6**, 39240; doi: 10.1038/srep39240 (2016).

**Publisher's note:** Springer Nature remains neutral with regard to jurisdictional claims in published maps and institutional affiliations.

## Supplementary Material

Supplementary Information
