# Supplementary material for: Gene expression in local stroma reflects breast tumor states and predicts patient outcome
Source: Sci Rep. 2016 Dec 16;6:39240. doi: 10.1038/srep39240 (PMC5159815; doi:10.1038/srep39240)
Supplement: Supplementary Information [file srep39240-s1.pdf]

Supplementary Information for *Gene expression in local stroma reflects breast tumor states and predicts patient outcome*

Russell Bainer[1]*, Casey Frankenberger[2]*, Daniel Rabe[2], Gary An[3], Yoav Gilad[1*] and Marsha Rich Rosner[2*]

[1]Department of Human Genetics, University of Chicago, Chicago, IL 60637, USA

[2]Ben May Department for Cancer Research, University of Chicago, Chicago, IL 60637, USA

[3]Department of Surgery, University of Chicago, Chicago, IL 60637, USA

*These authors contributed equally to this work

[#]Corresponding author. Email: m-rosner@uchicago.edu (M.R.); gliad@uchicago.edu (Y.G.)

The authors wish to take this opportunity to provide additional information regarding the implementation of the analyses discussed in the main text, and to explain the study in greater detail.

Motivation and Study Design Overview

The purpose of this experiment is to develop a suite of analytical tools for the computational deconvolution of mixed RNA samples derived from multiple species using RNAseq, and to apply these methods to rigorously characterize the local transcriptional changes that occur during tumor development in a xenograft model. This technique allows us to comprehensively survey transcription in developing tumors in parallel with the surrounding microenvironment *in vivo* at unprecedented levels of precision, allowing us to perform one of the first nearly comprehensive transcriptional analyses of the tumor microenvironment.

Specifically, we apply our method to a well-characterized model of metastasis suppression in order to identify genes whose transcription changes as a result of the presence of local primary tumors that do or do not express the RKIP metastasis suppressor. These genes are

likely to be involved in the metastasis-suppressive phenotype characteristic of RKIP expression and constitute strong candidates for followup study.

Briefly, the basic study design is as follows (further details follow below, and the entire study design is summarized in supplementary Figure 1 in the main text). We injected one rear mammary fat pad from each of five athymic nude mice with MDA-MB-231-derived BM1 breast carcinoma cells suspended in PBS and allowed tumors to develop for four weeks. We then sacrificed the mice and extracted both the tumor-bearing and uninjected fat pads, bisected each fat pad, and separately isolated RNA from each tissue section. We then sequenced these RNA samples using the Illumina HISEQ2000 platform and separated the mouse and human reads informatically to generate tumor-specific and stroma-specific expression estimates. Next, we compared these estimates with similarly-derived expression estimates from fat pads taken from mice injected with BM1 cells stably expressing the RKIP metastasis suppressor at physiological levels, and from mice that were sham injected and do not contain developing tumors, as well as from the BM1 cell cultures grown *in vitro*. These data allow us to identify both local and systemic changes in stromal expression related to the presence of developing invasive and noninvasive tumors, and to identify changes in tumor expression *in vivo* and *in vitro* without the confounding effect of transcription from infiltrating host cells.

Throughout the text below, we use “naïve injected fat pad” and “naïve uninjected fat pad” to refer to fat pads from animals that were sham injected with PBS that did not contain tumor cells. We use “uninjected fat pad” to refer to contralateral fat pads that were not injected with tumor cells but were taken from animals that contained developing tumors, and we use “tumor-associated stroma” to refer to fat pads that were directly injected with tumor cells and

contain developing tumors. We use "*in vitro* cell cultures" to refer to tumor cells grown in plates *in vitro* rather than within the xenograft context.

Cell Culture and Animal Protocols

All animal work was done in accordance with a protocol approved by the Institutional Animal Care and Use Committee. Stable bone metastatic MDA-MB-231 BM1 cells expressing Raf Kinase Inhibitory Protein (RKIP) were generated and maintained as previously described[1]. Briefly, the RKIP rescue cell line was generated by transducing target cells with HA–RKIP wt sub-cloned into a pCDH1-CMV-MCS1-EF1-copGFP lentiviral vector (System Biosciences, Mountain view, CA). Cells were grown in a complete medium consisting of DMEM supplemented with 10% FBS, 50 U/ml penicillin, and 50 μg/ml streptomycin.

All animal work was done in accordance with a protocol approved by the Institutional Animal Care and Use Committee. For tumor formation, cells were orthotopically injected (1×10^6 cells/0.1 ml) into the fourth mammary fat pad of anaesthetized athymic female nude mice. All mice were between six and seven weeks old, and were anaesthetized with a single injection containing 100 mg ketamine and 10 mg xylazine per kg. Four weeks following the injection, mice were anaesthetized with 2% isoflurane and injected intraperitoneally with D-luciferin (100 mg/kg in PBS) and imaged luciferase activity using an IVIS200 Imaging System (Xenogen) to confirm localized tumor development in the injected fat pad. The following day, mice were anaesthetized and the injected and noninjected rear mammary fat pads were surgically removed and immediately dropped into a tube containing 2ml RNAlater (Ambion). All mice were

sacrificed simultaneously, and instruments were cleaned in ethanol before removing each fat pad. Samples were incubated at 4°C overnight and then frozen at -80°C until RNA extraction.

RNA extraction, quality control, and sequencing library preparation

After thawing the samples on ice, we bisected the samples, dropped each fragment into lysis buffer, and homogenized the tissues using a Power Gen 125 homogenizer (Fisher). We then independently extracted RNA from each section using the RNEasy Mini kit (Qiagen) according to the manufacturer's protocol under RNAse-free conditions and performing the optional 5-minute on-column treatment with DNAse I to remove contaminating genomic DNA from the RNA sample. Total RNA was eluted in RNAse free water (Qiagen), and samples were aliquoted for analysis and stored at -80°C until further use. After ensuring that the extracted RNA samples were of high quality using an Agilent Bioanalyzer (RIN >= 8.5), we used the RNA to generate sequencing libraries using the Illumina Truseq RNA kit according to the manufacturer's protocol, and further size-selected each sample via polyacrylamide gel extraction using the MinElute Gel Extraction Mini Kit (Qiagen). The libraries were subsequently analyzed a second time using an Agilent Bioanalyzer to quantitate the library concentration and to verify that fragment sizes were consistent across samples prior to sequencing.

Massively Parallel Library Sequencing and Species-specific Read Disambiguation

Once we were satisfied that the libraries were of high quality, we sequenced all samples in multiplex (8 samples per lane) using an Illumina HISEQ2000 to generate 108bp single-end reads. We chose to generate long reads rather than paired-end reads because they contain more

sequence information and are consequently easier to separate by species origin. All samples from the tumor-injected mice were sequenced on the same flow cell with lanes assigned randomly to each sample, and a total of three flow cells were sequenced for the mouse samples with the lane assignments permuted. Technical replicates were never sequenced on the same lane. Sample libraries generated from the cell cultures and naïve fat pads were separately sequenced in multiplex one lane of a separate flow cell. Subsequent analysis of the expression data using nonparametric principal components analysis (PCA) did not detect systemic lane or flowcell-specific biases (data not shown).

Next, we recovered unassigned sample reads using a parsimony-based approach. In the multiplex sequencing reactions used in this experiment multiple samples are sequenced simultaneously on each lane of the flowcell, and the sequence of each cluster is assigned to its sample of origin by separately sequencing a 6-nucleotide barcode embedded in the adapter sequence. Specifically, after the sequence of each cluster is determined, the sequencing primer is eliminated from the flow chamber and a new indexing primer is introduced to sequence the barcode present in the adapter, and these barcodes are then used to parse the reads corresponding read sequences into individual samples. In cases where the sequenced indices did not match any of the indices present in the sample adapters (e.g., because of errors during the barcode sequencing reaction), we assigned reads to samples based on closest-match parsimony. Specifically, we assigned reads to the sample whose adapters most closely matched the observed index sequence. We discarded a read if its index differed from all of the sample indices at more than two locations, or if the observed sequence was equivalently distant from multiple sample index sequences. The number of ambiguous sequences recovered in this manner varied across

samples, but in all cases corresponded to < 1% of the total sequencing reads for that sample. This methodology was implemented in a perl script available upon request from RB.

Next, we separated sequencing reads in each sample by species origin. Our approach is conceptually similar to previously published methods, but modified slightly to improve recovery of annotated transcripts. First, we used bwa to align the raw *.fastq file to four sequence assemblies: the full set of human Refseq mRNA transcripts (hg19), the full set of mouse Refseq mRNA transcripts (mm9), the human genome (hg19), and the mouse genome (mm9). This resulted in four separate *.sai files for each sample, which we then converted to *.sam files using samtools. We then jointly analyzed the *.sam files to determine to which of the four assemblies each read could be aligned.

We used the following logic to partition the reads into genome-specific and transcriptome-specific transcription within each species based on the alignment information. If a read aligned uniquely (e.g., to a single location) to a species' genome assembly and could not be aligned to any of the other assemblies it was added to the genome-specific *.sam file for the appropriate species (see below). In cases where a read aligned uniquely to the transcriptome assembly in one species but did not align to any of the other assemblies, we considered the read transcriptome-specific and added the read to the transcriptome-specific *.sam file for the appropriate species. When a read could be aligned to both the transcriptome assembly and the genome assembly for one of the species and could not be aligned to either of the assemblies in the other species, we considered the read to be transcriptome-specific and added it to the corresponding *.sam file. Reads that could be aligned to any combination of assemblies from

both species, or which could be aligned to multiple genes or genomic regions within a species (see below) were considered ambiguous and removed from further analysis.

We used the transcriptome-specific *.sam files to estimate expression levels for each gene by counting the number of reads that were unambiguously aligned to each gene. In our analysis, regions within a given transcript may be functionally unalignable because of sequence similarity between species, and so both the overall length of each transcript and the expected distribution of reads across it is unknown. This prevents us from meaningfully estimating individual isoforms or splice variants, and we consequently chose to perform a gene-wise analysis by combining reads mapped to all known isoforms for a given gene into a single expression estimate. Specifically, we generated gene expression estimates as the number of reads that could only be aligned to transcript assemblies annotated to a single gene, even if the read could be aligned to multiple places within a transcript, or to multiple isoforms that were annotated to the same gene. These expression estimates were normalized and analyzed separately from the genome-specific reads, as detailed below.

To assess the performance of our read partitioning strategy, we combined sequencing read data obtained from libraries generated from human and mouse RNA samples in a previous experiment and applied the methodology described above. The data used in this simulation consisted of 13,342,415 human-derived and 17,087,540 mouse-derived 76bp sequences, originally sequenced on an Illumina GA II instrument. First, we appended the read labels within the human and mouse *.fastq files so that each read label explicitly indicated the sample of origin, and then combined the human and mouse reads into a single *.fastq file. We then aligned and partitioned this file as previously described. In this simulation, we were able to align

21,543,440 (70.8%) read sequences, consisting of 10,265,133 (76.9%) of the human-derived and 11,278,307 (66.0%) of the mouse-derived read sequences. Of the aligned sequences, 794,880 (7.7%) human and 527,024 (4.7%) mouse reads were aligned to both species and were discarded. 1,379 (0.013%) human reads were inappropriately assigned to the mouse alignment, and 9,197 (0.081%) mouse reads were similarly misassigned to the human genome, corresponding to an overall misassignment rate of 0.035%. It should also be noted that the bias towards successful alignment of human reads in both assemblies is expected because the mouse assembly is derived from the assembled human sequence, and because the human assembly is likely to be more accurate overall. Upon inspection the misassigned reads were overwhelmingly aligned to genomic regions annotated with multiple isoforms, paralogs, and/or evidence for alternative splicing, suggesting that the error rate is largely dependent on inconsistencies between the assembly sequence and the sequence present in the transcribed RNA. These genes and their orthologs were omitted from subsequent analyses (see below).

Overall, the sequencing read distributions observed in our experimental data were broadly consistent with those observed in the simulated dataset. Overall, we were able to unambiguously assign an average of 87.9% of all sequencing reads obtained within each sample. Among the samples containing RNA derived from only one species (e.g., human cell cultures and mouse sham-injected fat pads), of the successfully-aligned reads an average of 94.4% were aligned to the appropriate species of origin within each sample, with an average misassignment rate of 0.48% and 1.6% in the cell culture and fat pad samples, respectively. The percentage of reads successfully aligned to the human genome within the tumor-containing fat pads was variable across samples, ranging from 25.1% to 69.3% of the successfully aligned reads. Very

few human reads were observed in the uninjected fat pads across all tumor-bearing animals, with an average of 95.8% of all unambiguous reads aligning to the mouse assembly and 0.69% aligning to the human assembly. A summary of the read alignment data for all samples used in this experiment is included as **Supplementary Table S1**.

Estimation of Expression Levels

We used expression estimates derived from human-only (cell culture data) and mouse-only (naïve fat pad) samples to omit genes from our analysis for which we found evidence for alignment errors. Specifically, we counted the number of reads that unambiguously aligned to each gene within each species for each of the human-only and mouse-only samples, after combining the technically replicated cell line samples into single biological replicates and normalizing the total read counts within each sample such that the total number of reads uniquely aligned across all human and mouse transcripts was constant. We then compared the average number of reads uniquely aligned within each gene from the mouse-only and human-only samples, and excluded any genes where more than 5% of the aligned reads were derived from the incorrect species. This resulted in the exclusion of 1041 human and 26 mouse genes from subsequent analysis.

For the majority of the analyses described below, we used the total number of reads mapping to each gene within each tissue as the gene's expression estimate within a sample. We normalized the medians of these values across all samples by dividing the number of reads aligned to each gene in each sample by the ratio of the sample median to the mean of all similarly-calculated medians for all samples, and rounding the values to the nearest integer.

Tumor and stroma transcriptomes were normalized separately so as to remove biases related to the relative proportion of each tissue present in the sequenced samples. In analyses in which the length of a transcript is important (such as GOseq) we assigned genes functional transcript lengths, which correspond to the annotated transcript length weighted by its functional mappability in our experiment; further detail about the estimation of functional transcript lengths are included below.

Overall read assignment statistics for the data used in this experiment are summarized in **Supplementary Table S2**, and we note that the distribution of the read alignments among the samples included in the analysis (see below) are broadly consistent with the results of the simulation.

RNAseq expression quality control and selection of samples

After partitioning the transcriptomes and estimating expression levels as described above, we rigorously checked the expression data for consistency and to mitigate potential experimental biases. Unless otherwise noted, all of the following quality control procedures were performed on the transcript expression data. First, we analyzed the expression estimates in each sample and flowcell independently to determine whether there were detectable lane-specific or flowcell-specific effects in our data. In parallel analyses, we verified that the major results presented in this study are robust to the particular normalization methodology (quantile, RSN, InvariantSet; data not shown). We then confirmed that the major principal components of these data did not separate samples according to lane or flowcell assignment, and that expression estimates derived from the same libraries clustered hierarchically (via nonparametric PCA; data not shown). After

we were satisfied that our data were largely free of lane and flowcell effects, we combined the raw (unnormalized) data for each sample and normalized them by scaling the medians to produce sample gene-level expression estimates.

We first looked at broad signatures of the raw and normalized data to assess the consistency of the samples with the experimental parameters. For these analyses we focused on all genes in which there was no evidence for cross-species misalignment (described in the preceding section) and in which at least one read was uniquely aligned in all samples. We investigated the correlation structure of the data by generating pairwise Spearman Rank Correlation coefficients for each sample pair, and compared these statistics across and within experimental conditions (**Supplementary Figure S2**). The pairwise correlations were uniformly high. In the mouse samples, the highest correlations were observed between tissue replicate samples derived from the same mouse, followed by correlations between biological replicates taken from different mice, with the lowest correlations observed between fat pads that did or did not contain developing tumors. The human tumor samples were all tightly correlated within each replicate class, with major differences in correlation primarily related to *in vivo* vs. *in vitro* growth conditions.

We then used the sample pairwise correlation coefficients to hierarchically cluster the samples. When considering the mouse expression data, the samples generally clustered according to tumor presence or absence, with the exception of four of the fat pad samples (both fat pad samples from mouse 30, and the first fat pad replicate from mouse 26) from mice injected with BM1 tumor cells that clustered among the tumors. We subsequently performed principal components analysis on the expression data and found that these four samples were more closely

associated with the tumor-associated stroma samples with respect to the first and second principal components. Based on these data we felt that we could not exclude the possibility that these fat pads contained micrometastases that were not visible during the luciferase imaging and we removed them from subsequent analyses. After removing these outliers, all samples followed expected hierarchical clustering, PCA, and correlation structure patterns (**Supplementary Figures S2**).

After excluding these outliers, we used all of the samples in which we uniquely aligned at least 20 million reads in mice for which at least one tumor-associated stroma and one naïve fat pad sample were available for subsequent analyses. This resulted in a total of 19 (4 *in vitro* and 15 *in vivo*) human and 28 mouse samples (2 uninjected naïve fat pads, 2 injected naïve fat pads, 13 uninjected fat pads, and 11 tumor-associated stroma).

Analytical framework

To identify differences in gene expression related to tumor presence, we fit a set of gene-wise negative-binomially distributed linear models to the expression values observed for each gene across samples. In this analysis we model expression within each species independently and assume that the expression estimate for each gene (normalized number of uniquely aligned reads) follows a negative binomial distribution with mean μ and variance φ:

$$y = NB(\mu, \varphi)$$

In the mouse data, the subscript i indicates whether the mouse was injected with tumor cells or PBS, j indicates whether the fat pad contains a developing tumor, k indicates whether the

injected cell line expresses RKIP, l indicates the mouse of origin (biological replicate), and m indicates the technical replicate within each mouse.

We used all mouse samples to identify genes whose expression changes differently in the presence of RKIP-expressing tumors relative to the vector tumors by fitting the following model:

$$y_{ijklm} = NB(\mu_{ijklm}, \varphi)$$

Where

$$log(\mu_{ijklm}) = \beta + \psi_i + \tau_j + \psi\tau_{ij} + \tau\vartheta_{jk} + \psi\tau\omega_{ijk} + \theta_{ijkl} + \epsilon_{ijklm}$$

We fit this model to each gene, and all coefficients correspond to fixed effects. β corresponds to the intercept, ψ is a term capturing global changes in gene expression related to whether the fat pad contains the injection site, τ estimates the systemic effect of the developing tumor on the gene's expression level in the uninjected fat pad, the interaction effect ψτ captures expression changes at the injection site that are specifically related to tumor development, the interaction term $\tau\vartheta$ captures differences in the gene's expression level between uninjected fat pads in mice containing tumors that do or do not express RKIP, and $\psi\tau\omega$ is an interaction term similarly capturing differences in expression between injected fat pads in mice bearing RKIP-expressing or vector-expressing tumors. θ captures biological variance across mice, and ε corresponds to the residual error, assumed to be Normally distributed with mean zero and variance s_ε^2.

We fit the model to each gene using an alternating iterative maximum likelihood estimation approach implemented in the glm.nb function in the R package MASS. In cases where the overdispersion parameter was estimated to be very large ($\varphi > 1500$) we assumed that the

model does not fit the data well and excluded the corresponding gene from further analysis.

We used this model to identify discordant differences in mouse gene expression related to RKIP expression by comparing the following hypotheses:

H^0: $\beta \neq 0, \psi^i \neq 0, \tau^j \neq 0, \psi\tau^{ij} \neq 0, \tau\rho^{jk} \neq 0, \psi\tau\rho^{ijk} \neq 0, \theta^{ijkl} \neq 0$

H^1: $\beta \neq 0, \psi^i \neq 0, \tau^j \neq 0, \psi\tau^{ij} \neq 0, \tau\varsigma^{jk} \neq 0, \psi\tau\omega^{ijk} = 0, \theta^{ijkl} \neq 0$

H_0 represents the full model, in which the means within each class are estimated independently. To identify genes that are discordantly differentially expressed between the uninjected fat pads and fat pads that contain developing tumors in mice with or without RKIP expression in the primary lesion, we compared the full model to reduced model H_1, in which we assume that we assume that the gene is similarly expressed in tumor-associated stroma regardless of RKIP expression levels in the primary tumor. We assessed the significance to the observed differential expression in the framework of the generalized linear model using a Wald test, and corrected p-values for multiple tests using the FDR approach described elsewhere.

Ontological Enrichment Analysis

It has been shown that the power to detect gene expression differences in RNAseq data is influenced by the size of the gene being analyzed, which can affect ontological enrichment analyses. In our data, the functional gene length (i.e., the size of the unambiguously mappable portion of the gene) is unknown *a priori*, so we estimated these values as follows. We generated human-specific and mouse-specific *.fastq files containing artificial sequencing reads tiling the

corresponding reference transcript sequences, such that the file contained one read for each nucleotide position present in the reference sequence, where successive reads are offset by one base pair from the previous one. We then aligned and partitioned these files as described above, and calculated the per-base mappability within each reference sequence as the mean proportion of successfully mapped bases at each location within each transcript. We then multiplied the mean per-base mappability of each transcript by the length of the transcript reference to generate a functional transcript length, and then used the median of the functional transcript lengths of each transcript annotated to each gene as the functional gene length. We used these lengths to calculate the appropriate genewise probability weighting function (PWF) using the nullp function implemented in the GoSeq R package[9]. We subsequently used this PWF to weight *p*-values for subsequent ontological enrichment analyses implemented in GoSeq, and during the cell type enrichment analyses below.

To perform ontological enrichment analyses we compared the specified set of significant genes to the total set of successfully-modeled genes in the corresponding dataset. We identified enriched categories with the goseq function implemented in GoSeq using the default settings (Wallenius approximation), and corrected p-values for multiple testing using the Benjamini and Hochberg approach.

We used GOseq to determine the ontological categories enriched within sets of genes whose boundaries may be clearly defined (e.g., differential expression at an FDR of 0.01). For statistics that are continuous and in which appropriate cutoff values are less clear, such as correlation coefficients, we used GSEA with the statistics considered as a ranked list using the default settings.

WGCNA

Individual gene homolog pairs in human and mouse were identified using the Homologene database release 65 by using the Homologene Matcher tool implemented on the RefDIC database.

We performed whole genome coexpression network analysis using the expression estimates of 11,181 genes for which the human gene was unambiguously associated with a single mouse homolog, and for which the mouse homolog was unambiguously assigned to the corresponding human gene. To perform the analysis we generated two matrices of gene expression estimates from the set of samples for which both tumor and stroma expression estimates were available, maintaining the sample order between mouse-specific and human specific matrices. Each matrix contained data from eleven paired tumor-stroma samples (5 containing control tumors and 6 containing RKIP-expressing tumors). WGCNA assembles modules based on parametric correlation estimates, so we used the log-transformed number of reads aligned to each gene, converted to FPKM as the expression estimate. We then independently generated tumor and stroma coexpression networks from the gene data, and extracted the eigengenes from each of the resulting modules. We subsequently generated a correlation matrix using these eigengenes, and identified pairs of coexpressed modules based on the pairwise Pearson's correlation coefficients generated by comparing the eigengenes of the mouse and human expression modules. All reported module pairs contain disproportionately high numbers of homologous genes ($P = 9.0 \times 10^{-8}$, $P = 4.0 \times 10^{-5}$, $P = 0.02$, Fisher's exact test), and were not apparently affected by misalignment (correlation coefficients are not well

correlated with misalignment rates). We estimated ontological enrichment within the modules using goseq as described above, using the set of homologs present in both modules.

Clustering Analysis of Human Breast Cancer Samples

To determine whether the genes identified in our analysis could be used to improve classification of breast cancer patient samples, we employed a metric similar to the nearest-neighbor clustering approach described previously. First, we hierarchically clustered the patient samples on the basis of their euclidean distance using the `hclust` function implemented in R (http:www.r-project.org). Then, we constructed a dendrogram in which each sample is assigned to a leaf, and we defined the extent to which samples of a given Pam50 subtype were correctly clustered as the proportion of all nearest-neighbor leaves of all samples in the group that also contained samples of the same Pam50 subtype. The leftmost and rightmost leaves on the dendrogram were assumed to have one nearest neighbor, and all internal leaves have two. Thus, within each dataset each Pam50 subtype was assigned a nearest-neighbor clustering score between zero and one, such that subtypes with a score of one are clustered into a single uninterrupted bloc, and a subtype with a score of zero contains samples that never cluster next to each other.

To test the ability of each gene set to improve clustering of a given subtype, we compared the clustering score assigned to that subtype to the distribution of clustering scores similarly generated using the expression levels of 10,000 randomly selected sets of genes of the same size as the true set. We defined empirical p-values as the proportion of random gene sets which produced an equal or higher clustering score than that observed for the subset in the real data. As

a multiple test correction, we performed five permutations within each of the 10,000 iterations and selected the highest clustering score to build the corresponding null distribution.

Real time qPCR Validation of Local and Distal Gene Expression

In an independent panel of mice, we injected one rear mammary fat pad from each of five athymic nude mice with MDA-MB-231-derived BM1 breast carcinoma cells suspended in PBS and five athymic nude mice with MDA-MB-231-derived BM1 expressing the metastasis suppressor RKIP and allowed tumors to develop for four weeks. We then sacrificed the mice and extracted both the tumor-bearing and uninjected fat pads, bisected each fat pad, and isolated RNA from local tumor and fat pad combined and from contralateral fat pad separately. Species specific primers were generated to estimate expression levels in combined human and mouse tissue samples. Primers were generated to test expression levels in RNA purified from distal fat pads.

Human gene specific primers:

Beta Actin : Forward Primer tggcaccacaccttctacaa, Reverse Primer ccagaggcgtacagggatag

MCPH1: Forward Primer atgtagtggcctatgttgaagtg, Reverse Primer ccacaagctgtgttgtaaatgtc

SLK: Forward Primer gccacctacattagcacagc, Reverse Primer tctcggatgggtttgttgga

DEXI: Forward Primer cctgtactacgccttcctca, Reverse Primer ccaagtacgcatcaaagacg

Mouse gene specific primers:

Rpl4: Forward Primer gcccagaaatccaaagagcc, Reverse Primer cttctcggatttggttgcca

Mcph1: Forward Primer tctgaaagatgttgtggcctatg, Reverse Primer ggtcacttgcttgttcaaggtt

Slk: Forward Primer gcattgccagctctgaagaa, Reverse Primer cacttgcatgctcatccaca

Dexi: Forward Primer aaccagacgtcacctctctg, Reverse Primer tcctcagagtgactccctga

Validation of Tumor Stroma Gene Expression Correlation in Patient Data

To test whether gene expression levels are correlated in tumor and stroma tissues derived from human patients we used an expression microarray dataset deposited in the Gene Expression Omnibus by Boersma et al. (GSE5847). The data set consists of 90 microarrays hybridized with RNA from paired tumor and stroma samples isolated via laser capture microdissection from inflammatory and non-inflammatory breast cancer samples. We classified the tumor tissues into basal, HER2 positive, normal, and luminal (combining lunminal A and luminal B) subtypes using the PAM50 classifier. We then calculated the Spearman correlation coefficient relating the expression estimate of each probe set in the tumor and stroma tissues, and plotted the distribution of these values for all samples or calculating them separately with only the basal and luminal, or inflammatory and non-inflammatory patient subsets.

Survival Analysis

To determine if the expression levels of an identified set of genes were prognostic for metastasis-free survival in human patient data, we used the rsf function implemented in the randomSurvivalForest R package to assign variable importance scores to each gene in that set on the basis of their expression within the human breast cancer data set. We combined all genes with positive variable importance scores into a classifier, which we used to subdivide the patient samples into two groups via k-means clustering. Finally, we determined whether there was a relationship between group assignment and metastasis-free survival by fitting a cox proportional

hazards model to the patient data and including histological subtype and patient age as covariates. We tested the significance of the association in the framework of the model using a Wald test.

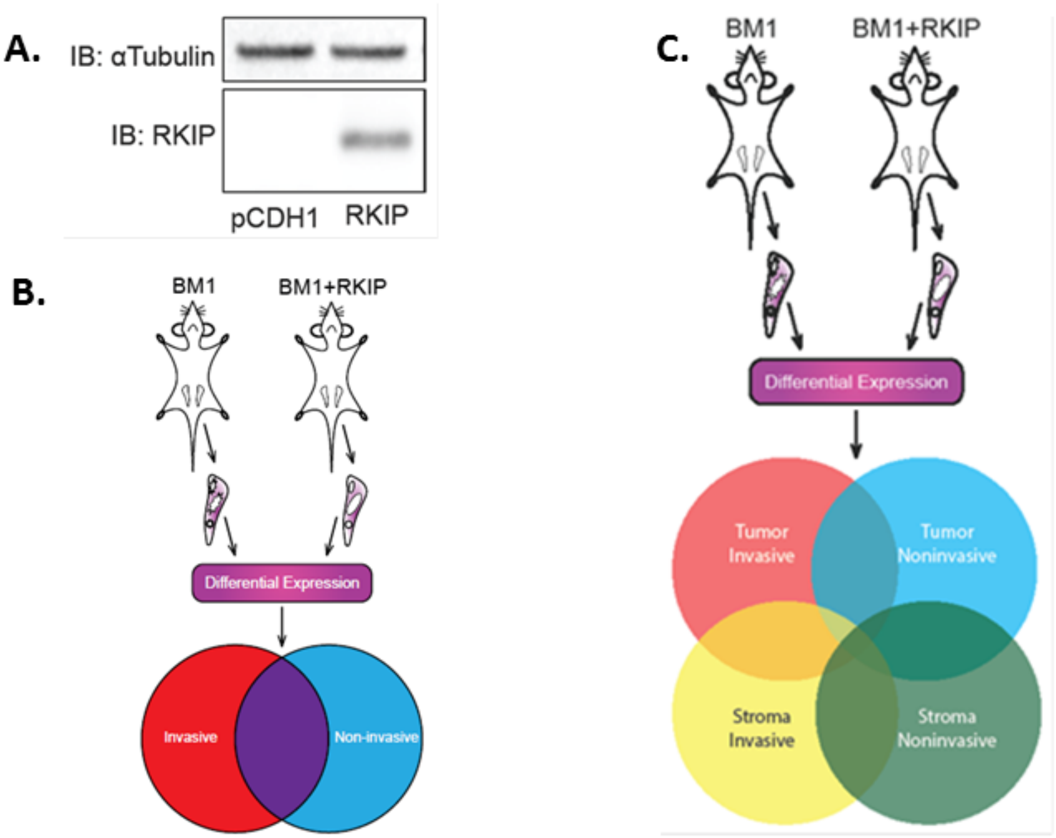

Supplementary Figure S1. A) Validation of RKIP expression in MDA-MB-231BM1 cells. Protein lysate from bonetropic metastatic MDA-MB-231 derived triple negative breast cancer 1833 BM1 cell lines expressing pCDH1 vector (left) or wild-type RKIP (right) were immunoblotted with RKIP and α-tubulin antibodies. B) Scheme depicting comparison between tumor and stroma-derived mRNA expression levels from isogenic TNBC xenograft models in which the metastatic phenotype is suppressed by exogenous expression of RKIP. C) Scheme illustrating separation of sequence reads on the basis of species origin derived from whole mammary fat pads containing developing tumors. Human- and mouse-derived reads are shown in red and blue, respectively.

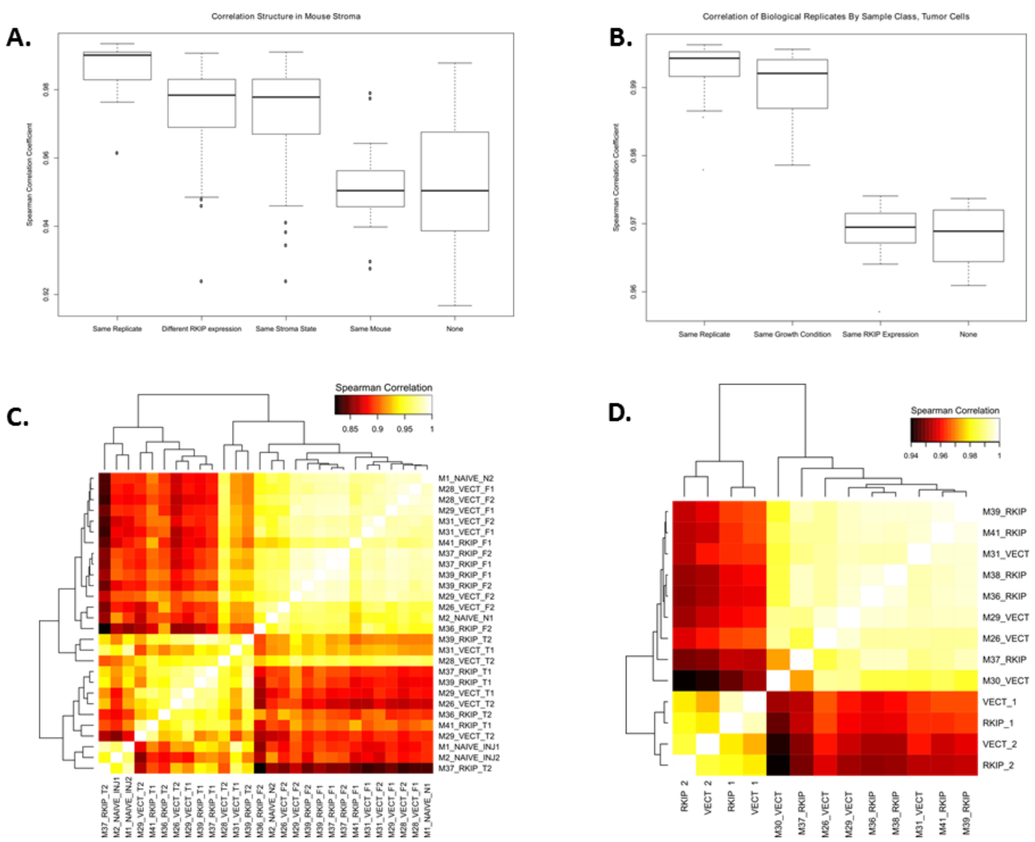

Supplementary Figure S2. A) Boxplots of Stroma Gene Expression Correlation Structure. Boxplots summarizing the distributions of Spearman pairwise correlation coefficients comparing (left to right): Same Replicate: all replicates taken from the same sample group (fat pad or TAS) within the same animal; Different RKIP Expression: samples of the same sample group but contrasting mice injected with metastatic and nonmetastatic tumors; Same Stroma State: samples of the same sample group but contrasting mice injected with PBS, metastatic, and nonmetastatic tumors; Same Mouse: All samples taken from within the same mice, regardless of injection or tumor presence; None: samples from different sample groups in different mice. B) Boxplots of Tumor Gene Expression Correlation Structure. Boxplots summarizing the distributions of Spearman pairwise correlation coefficients comparing (left to right): Same Replicate: all replicate samples sharing the same RKIP expression state and growth condition (e.g., *in vitro* or *in vivo*); Same Growth Condition: all samples sharing the same growth condition but in which RKIP is expressed at different levels; Same RKIP Expression: all samples sharing the same RKIP expression state in different growth conditions; None: all samples differing in both RKIP expression level and growth condition. C) Pairwise Spearman Correlation Matrix of Gene Expression Estimates in Stroma. Heatmap of all stromal samples clustered by their pairwise spearman correlation coefficients. Row and column labels show the Mouse, injection type (VECT: metastatic cells, RKIP: nonmetastatic cells, NAÏVE: PBS-injected) and stroma state (F: uninjected fat pad, T: tumor-associated stroma, N: uninjected fat pad in mock-treated mice, INJ: fat pad containing PBS injection site). D) Pairwise Spearman Correlation Matrix of Gene

Expression Estimates in Tumor Cells. Heatmap of all tumor samples clustered by their pairwise spearman correlation coefficients. Row and column labels show the mouse and RKIP expression status for *in vivo* tumor cells (VECT: metastatic cells, RKIP: nonmetastatic cells), or the RKIP expression status and replicate number of cells grown *in vitro*.

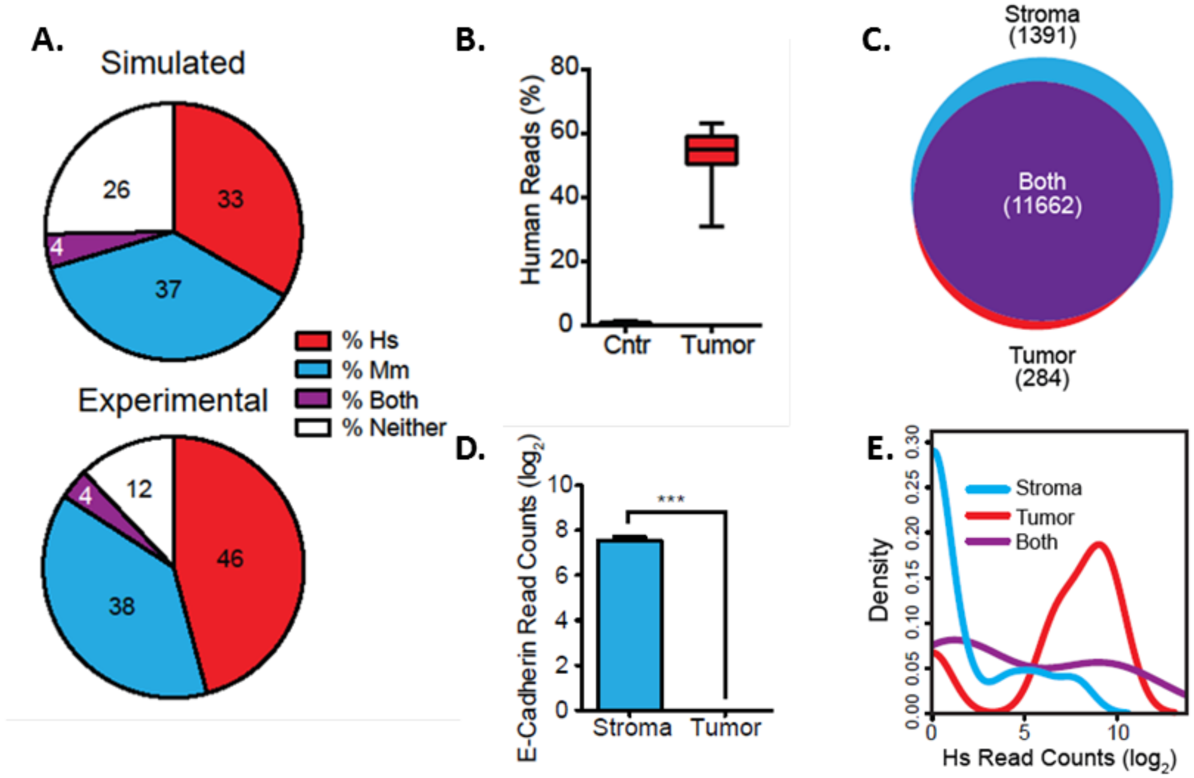

Supplementary Figure S3. A) The majority of sequencing reads can be accurately assigned to tumor and stroma tissues on the basis of sequence content, both in simulated xenograft RNAseq experiments (76bp reads, top) or in samples derived from direct sequencing of xenograft tissue (105bp reads, bottom). B) Human sequencing reads are largely absent from fat pads that do (tumor) or do not (contr) contain tumors. C) Most genes with unambiguous homologs in both species can be detected in both tumor and stroma tissue (one or more reads). D) E-cadherin is exclusively expressed in the stroma, mRNA expression is quantified as the total number of reads aligned to all transcript isoforms annotated to the corresponding human or mouse gene. E) Distribution of expression levels in tumor tissue of mRNA encoding ECM proteins originating from human tumors (red), mouse stroma (blue), or both tissues (purple). Tissue origin of ECM proteins was assigned on the basis of tandem mass spectrometry in a different xenograft model; density (y-axis) reflects the frequency of gene expression at the corresponding expression level within each group.

A.

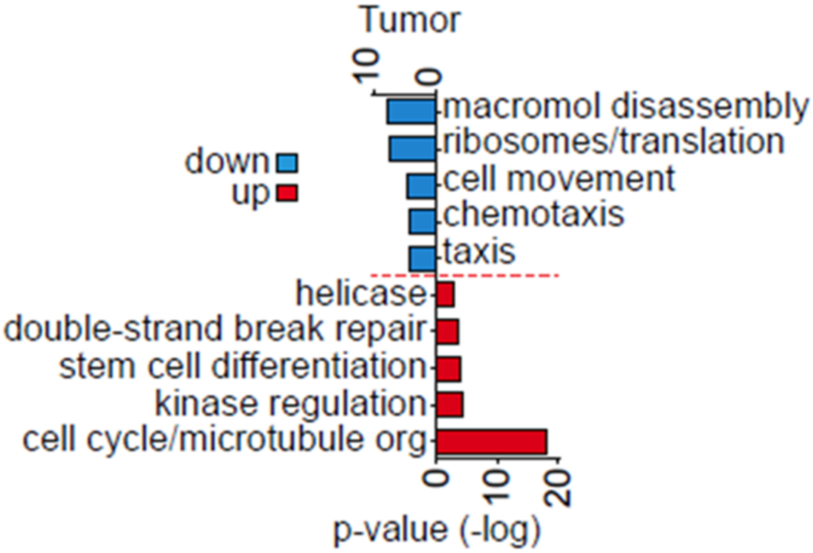

Supplementary Figure S4. A) Ontological categories enriched among genes whose expression level is significantly increased (red) or decreased (blue) in metastatic (RKIP-) relative to nonmetastatic (RKIP+) tumors. (FDR = 0.01 for all comparisons). Horizontal bars indicate the -log10 enrichment p-value.

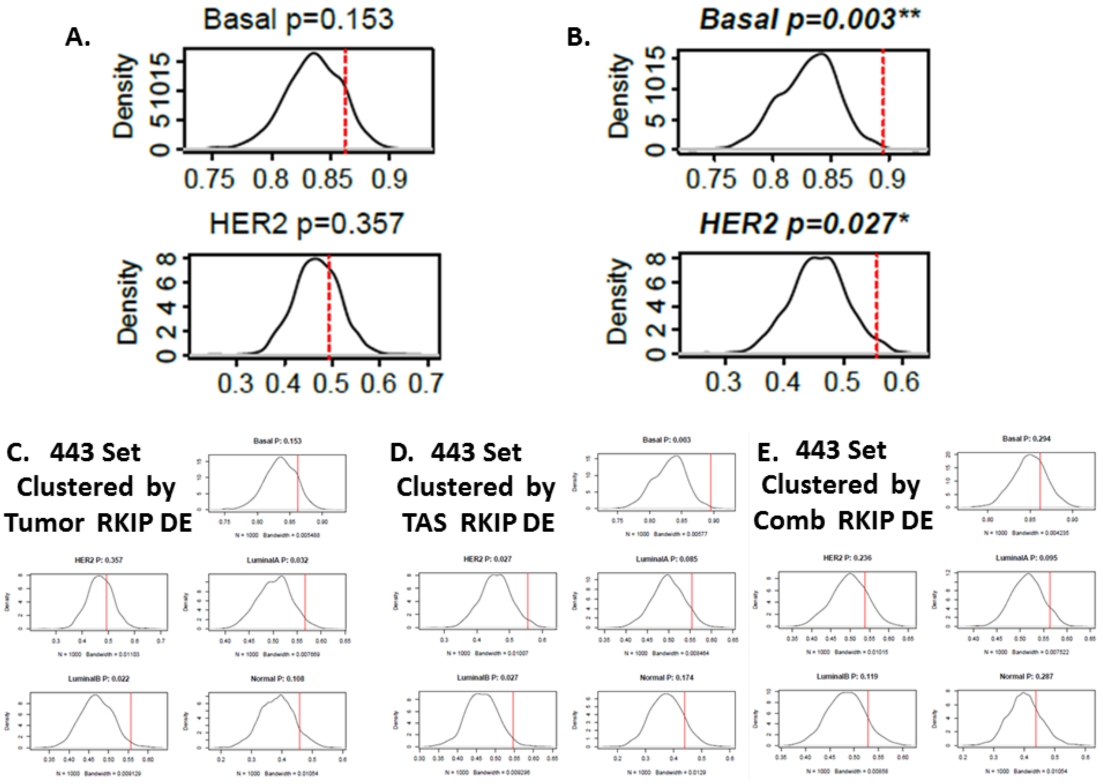

Supplementary Figure S5. Comparing genes differentially expressed between metastatic and nonmetastatic in A) human tumors or B) mouse stroma (FDR = 0.01) mouse stroma enable superior clustering of human Her2 and Basal tumor samples relative to arbitrary gene sets and genes differentially expressed between metastatic and nonmetastatic tumor tissue. In each plot, observed nearest-neighbor clustering scores (x-axis, red line) are contrasted with the distribution of scores derived from permuted gene lists (black line). C) Complete set of nearest-neighbor clustering in 443 patients using tumor DE genes to classify. D) Complete set of nearest-neighbor clustering in 443 patients using stroma DE genes to classify. E) Complete set of nearest-neighbor clustering in 443 patients using tumor and stroma combined set of DE genes to classify.

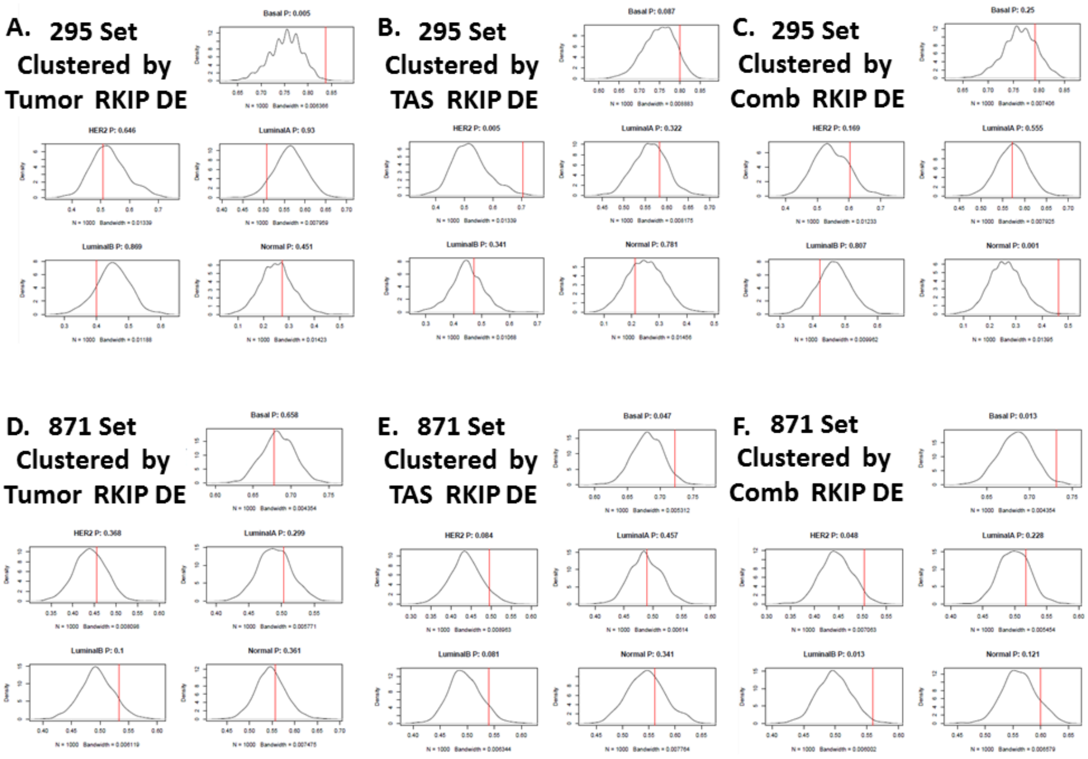

Supplementary Figure S6. A) Complete set of nearest-neighbor clustering in 295 patients using tumor DE genes to classify. B) Complete set of nearest-neighbor clustering in 295 patients using stroma DE genes to classify. C) Complete set of nearest-neighbor clustering in 295 patients using tumor and stroma combined set of DE genes to classify. D) Complete set of nearest-neighbor clustering in 871 patients using tumor DE genes to classify. E) Complete set of nearest-neighbor clustering in 871 patients using stroma DE genes to classify. F) Complete set of nearest-neighbor clustering in 871 patients using tumor and stroma combined set of DE genes to classify.

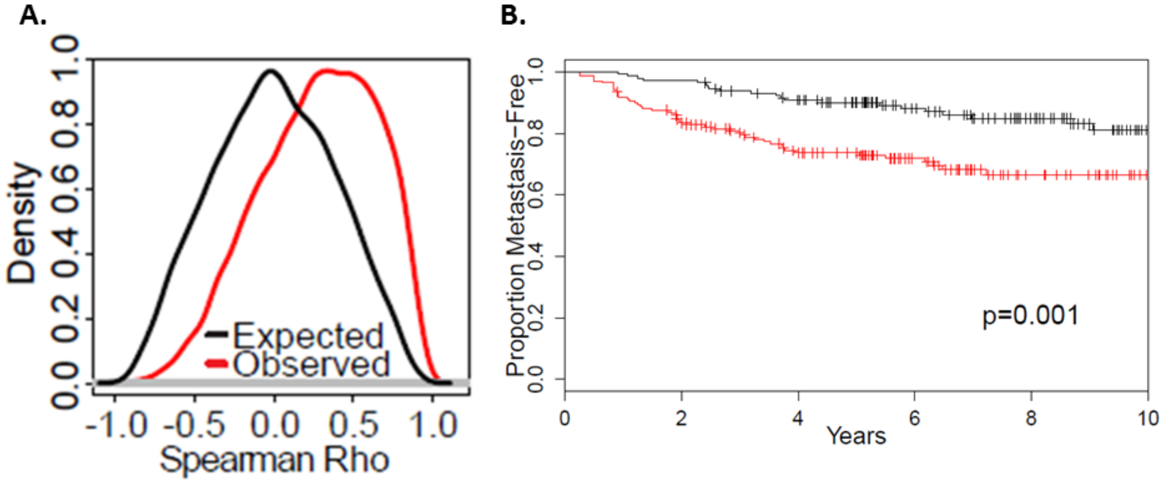

SupplementaryFigure S7. A) Density of pairwise Spearman coefficient estimates summarizing the correlation between tumor and stroma mRNA expression levels (red) for all unambiguous gene ortholog pairs. A permutation-derived null distribution is indicated in black. B) Kaplan-Meier plot demonstrating improved prediction of metastasis-free survival (MFS, y-axis) in a second validation set of 341 human breast cancer patients by a classifier derived from genes mRNA expression is inversely correlated between tumor and stroma (see above and main text, red line). After stratifying patients on the basis of the classifier, we estimated the significance of the association with MFS in the framework of a Cox multivariate regression model using a Wald test to generate P-values.

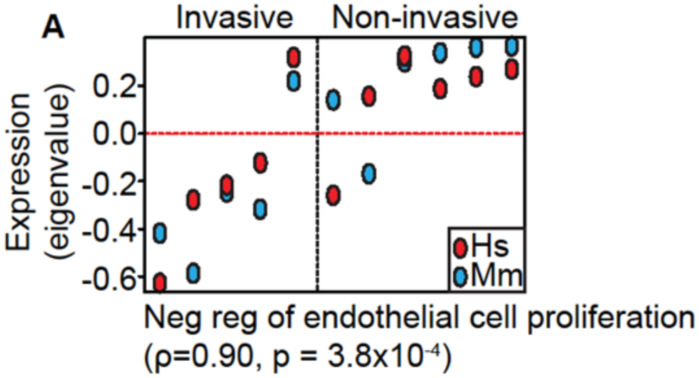

Figure S8. A) Additional example of correlated gene expression modules in tumor and adjacent stroma tissue. Eigengene expression values (eigenvalues, y-axis) derived from WGCNA analysis of tumor (red) and stroma (blue) tissues are indicated for all tumor samples analyzed (x-axis; individual tumors are distributed along the x-axis and stratified by metastatic (RKIP-) and nonmetastatic (RKIP+) tumor type). Module eigengene spearman correlation coefficients and the most enriched ontological category among the genes whose homologs are present in both modules are indicated below the plot; *P*-value indicates the nominal significance of the ontological enrichment. B) Validation of correlated gene expression in an independent group of BM1 xenograft tumors via qRT-PCR. Representative genes from the modules above were assayed with species-specific PCR primer sets, and expression estimates normalized to human *GAPDH* or mouse *Rpl4* are indicated on the x and y axes (ΔΔCt method). Spearman correlation coefficients and corresponding p-values (via permutation) are indicated.

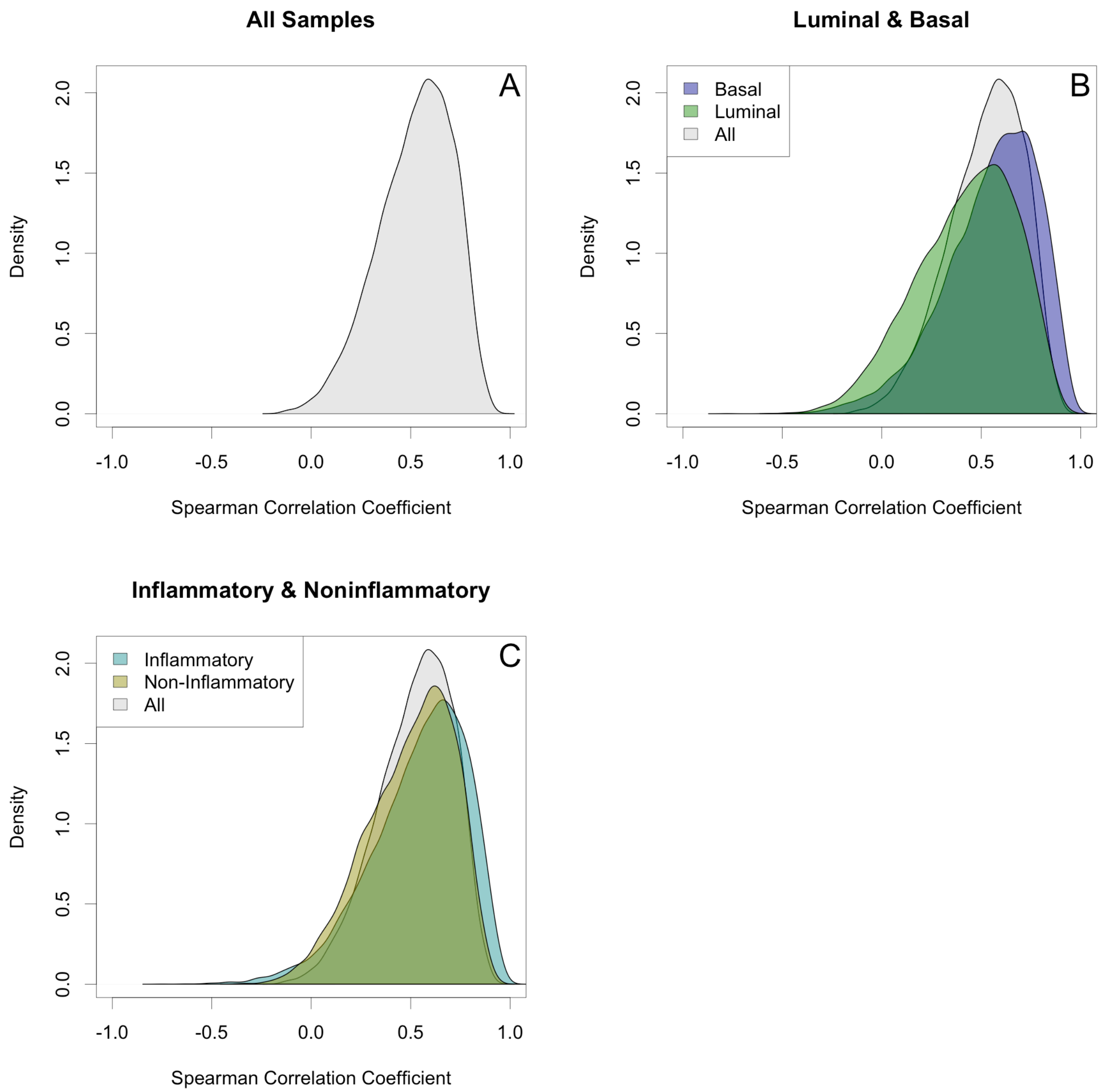

Figure S9. Pairwise correlation of tumor and stroma gene expression in GSE5847 by tumor subtype. Each panel depicts the density of spearman correlation coefficients calculated pairwise between the expression estimates of microarray probes hybridized with human tumor and paired stroma tissues isolated via laser capture microdissection. A) Density of pairwise coefficients for all 46 samples included in the study. B) As in A, but superimposed with densities derived from only luminal-type ($N = 23$, green) and basal-type ($N = 13$, blue) tumors as defined by the PAM50 classifier. C) As in A, but superimposed with densities derived from only inflammatory ($N = 12$, yellow) and non-inflammatory ($N = 34$, blue) tumor samples.

Supplementary Table S1. Sequencing read partitioning statistics by sample. Read counts are shown in millions.

RKIP	Mouse 36				Mouse 37				Mouse 38				Mouse 39				Mouse 41			
	F1	F2	T1	T2	F1	F2	T1	T2	F1	F2	T1	T2	F1	F2	T1	T2	F1	F2	T1	T2
Reads	28.52339	33.66205	75.7208	64.14651	32.51614	37.17858	122.3405	52.30696	65.90784	25.40124	50.72372	84.72839	49.68873	37.16206	108.8539	77.65757	42.77469	39.59814	97.0111	59.99148
HS	0.198499	0.064578	44.3136	31.94213	0.054188	0.037685	74.20233	0.101288	17.92416	0.067683	25.24783	42.04928	0.663808	0.12822	55.04324	40.5735	0.191395	0.194131	49.8024	36.81151
MM	23.38519	28.62624	18.57732	22.59841	27.75182	31.74928	28.12488	43.22529	37.72201	21.56832	17.36468	29.48344	41.47191	31.55355	36.02074	23.63296	36.10911	33.80202	31.45066	13.89498
Both	0.898025	1.137749	2.927019	2.623784	0.929766	1.130949	4.687435	1.922683	2.038304	0.871272	1.94785	3.175472	1.386511	1.204591	4.190803	2.973939	1.201447	1.416927	4.006262	2.456994
NoMap	4.041679	3.83349	9.902855	6.982188	3.78037	4.260672	15.32582	7.057694	8.223362	2.893965	6.163374	10.0202	6.166497	4.275703	13.59913	10.47717	5.272742	4.185062	11.75178	6.827995

Vector	Mouse 26				Mouse 28				Mouse 29				Mouse 30				Mouse 31			
	F1	F2	T1	T2	F1	F2	T1	T2	F1	F2	T1	T2	F1	F2	T1	T2	F1	F2	T1	T2
Reads	45.93758	31.91989	54.16869	91.02296	35.45746	37.62033	75.0697	68.48256	48.80632	44.1022	120.1046	59.34075	34.09069	54.93995	49.32073	79.45638	43.12573	35.59516	33.30568	69.64137
HS	0.086151	0.084154	28.8834	53.6655	0.37183	0.220994	2.926618	14.99536	0.402608	0.64798	73.77065	29.41756	0.25565	1.089642	11.46372	24.6687	0.274583	0.208119	1.127123	44.05236
MM	38.36367	27.12058	16.86814	22.74665	29.8223	31.61635	62.26497	42.38243	41.12692	36.81223	27.09654	20.98764	28.17961	45.48642	30.27967	43.27227	36.30472	30.41894	26.54422	14.44842
Both	1.487607	1.198044	2.053979	3.710218	0.999482	1.089972	2.291767	2.295147	1.311513	1.329616	4.722762	2.444885	1.120149	1.894048	1.797296	2.756828	1.212955	1.194383	1.345918	2.844826
NoMap	6.000157	3.517118	6.363174	10.9006	4.263844	4.693021	7.58635	8.809619	5.965278	5.312368	14.51465	6.490669	4.535284	6.46984	5.780058	8.758587	5.333468	3.773718	4.28841	8.295767

Cell Lines	RKIP1	RKIP2	PCP1	PCP2
Reads	24.11107	18.99719	31.49668	16.40304
HS	21.123	16.36458	27.44139	14.13646
MM	0.087676	0.076511	0.137092	0.093877
Both	1.093834	0.957302	1.430683	0.759371
NoMap	1.806561	1.598798	2.487524	1.413334

PBS	NAIVE_1	NAIVE_2	INJECT_1	INJECT_2
Reads	36.85891	27.73334	18.34627	21.06331
HS	0.913771	0.285331	0.187915	0.259488
MM	31.618	23.65247	15.1268	16.80384
Both	1.177015	0.98586	0.716081	0.763685
NoMap	3.150128	2.809688	2.315477	3.2363

F: Uninected fat pad sample; T: Tumor-bearing sample; NAÏVE: uninjected fat pad in mouse injected with PBS; INJECT: Fat pad containing injection site in mouse injected with PBS

HS: Human-aligned reads; MM: Mouse-aligned reads; Both: Ambiguous reads; NoMap: Unalignable reads.

Grey backgrounds and red text indicate stroma and tumor samples excluded from the analysis on the basis of coverage or sample clustering, respectively.

Supplementary Table S2. Genes used in Gene Signatures for stratifying breast cancer patient Metastasis Free Survival.

Genes Used in MFS Signatures			
Dist-TAS Common		Neg CoExpr	
SEPT9		BMP1	
ANXA9		C1orf109	
ARFGAP1		C6orf162	
BTBD9		CD72	
CD24		COL18A1	
CHST7		CPM	
CRY1		DRP2	
CTSK		EMILIN2	
DNASE1L2		FXC1	
DOLPP1		GAB1	
DSCAM		GTF2H2	
EPS8L1		HSP90B1	
ESM1		LAMA2	
F3		MAPKAPK3	
FAM63B		METTL8	
FBN2		MTRF1L	
FUS		PAQR5	
GPR126		PCDH1	
HMGB3		PCSK6	
IL8		PHF16	
KIAA0355		RHBDL2	
KIAA0556		SLC29A3	
KIF5C		SLC6A9	
KRT34		SP100	
LRRC1		SRPK1	
MAGEH1		TAF4B	
MUC1		THUMPD1	
MYB		ZNF35	
NR1D1			
NSDHL			
P2RX5			
PELO			
PTN			
PYCR1			
RABL3			
RGS2			
RPL30			
SERINC3			
SLC25A15			
SLC25A22			
SLC46A3			
SLC6A4			
TBC1D12			
TMEM158			
TNFSF18			
